# Active Gaming as a Mechanism to Promote Physical Activity and Fundamental Movement Skill in Children

**DOI:** 10.3389/fpubh.2013.00074

**Published:** 2013-12-24

**Authors:** Lisa M. Barnett, Shaun Bangay, Sophie McKenzie, Nicola D. Ridgers

**Affiliations:** ^1^School of Health and Social Development, Deakin University, Melbourne, VIC, Australia; ^2^School of Information Technology, Deakin University, Melbourne, VIC, Australia; ^3^Centre for Physical Activity and Nutrition Research, Deakin University, Melbourne, VIC, Australia

**Keywords:** motor skill, augmented reality, active video games

Insufficient physical activity is a global health issue (1). Australian physical activity guidelines recommend children engage in 60 min or more of moderate- to vigorous-intensity physical activity every day (2). In 2011–2012, only 19% of Australian children met these recommendations (3). Fundamental movement skills (FMS; e.g., run, jump, catch, kick, hop) are the building blocks of physical activity and underpin successful participation in sports games. FMS are not, however, naturally acquired (4). Less than 50% of Australian school aged children demonstrate competence of key FMS (5, 6) and trends across the last decade are no better (7).

Low levels of physical activity and FMS are coupled with ubiquitous electronic media use in the Australian home. Nearly all households with children (98%) have access to computer games (8). By 2014, it is predicted 87% of 6–10 year olds and 96% of 11–15 year olds will be playing computer games, with sessions typically occurring every 2 days and ranging in duration from half an hour (6–10 year old girls) to over 2 h (in 11–15 year old boys) (8). Incentives for game play include playing for the fun, challenge, and stimulation, with exercise benefits rated last (8). Since the chances of removing screen based behavior from children’s twenty-first century lives is virtually nil, developments that integrate computer gaming with opportunities for physical activity and FMS may elicit beneficial changes in these outcomes and overall health.

A strategy for introducing physical activity into gaming has been through the development of active video games (AVG). Technologies such as the Microsoft Kinect and Nintendo Wii deploy sophisticated controllers which sense whole body motion through depth cameras, accelerometers, and pressure sensors. However, these are constrained to a finite but controlled indoor environment which may limit opportunities for physical activity and FMS practice. For example, whilst review evidence suggests AVG play can result in light-to-moderate intensity physical activity (9, 10), it is likely the modest physical activity provision is inadequate for helping children meet national recommendations (11, 12). There is some evidence that AVG play is associated with higher movement skill proficiency in young children (13), and while children believe they are developing skill (14), few demonstrable skill components have been observed (15).

An interesting development in AVG play are mobile active games that allow game play to become pervasive (through continuous tracking via a personal mobile device) (16), accumulative (encouraging multiple informal physical activities on an ongoing basis) (17), and persistent (through logging and scoring over an extended duration) (18), whilst removing game constraints that limit games to a fixed location. Smartphones are particularly universal devices that can be used to support and facilitate mobile active games, especially as they are well integrated into an individual’s daily activities, have sufficient processing capability to present a personalized experience to the user (19), and can utilize a range of in-built sensors (e.g., WiFi, GPS) and features (e.g., audio, camera).

An example of AVG play using smartphones is through overlaying synthetic visual content above images of the real world (augmented reality; AR). Existing AR mobile applications fall into two categories. In the first, specially designed reference images are provided as posters or cards in the physical world. Digital content is generated relative to these reference points with the combined result shown on the mobile device’s screen. Live processing of the video stream from the camera allows control of the overlaid digital images. For example, the mobile game “Rolling Dead” overlays virtual zombies over a view of a player controlled robotic ball viewed through the camera and screen of the mobile phone (accessed 7/11/2013)^1^. Several AR games use sport inspired themes, such as AR Soccer (accessed on 7/11/2013)^2^.

The second approach employs location-based gaming, which exploits position tracking of the player through GPS and other location measures. Location sensitive games provide a novel yet underutilized opportunity to promote physical activity and FMS in children whilst achieving game outcomes. For example, children can engage in active yet unrelated movements to earn game play time (20) and to correctly perform exercises (21, 22). Indeed, general physical activity during play can be encouraged and rewarded (23). The game elements introduced through AVG provide persuasive motivation to continue the activity long enough to gain benefit from the physical activity (16, 19, 24, 25), provide purposeful action in support of narrative and game mechanics (26), and encourage thinking and problem solving skills at the same time as physical activity (27).

Whilst there are benefits to such techniques, limitations do exist. For example, many active mobile games distinguish activities using only thresholds on acceleration variance and device orientation (18, 21), or classifiers such as artificial neural networks or hidden Markov models (17). Misclassification or failing to register valid actions is significantly de-motivational (19), particularly if the player is erroneously reprimanded. The designers of mobile games must consider these issues if they are to use different sensor perceptions to encourage engagement in physically active behaviors in the physical environment.

To date, few studies have examined the use of location-based games in the promotion of physical activity. Suggestions for design elements to motivate physical activity in games include music integration, facilitation of short and long term fitness goals, and the ability to form groups, though these have not been validated (27). One study in children explicitly attempted to build in FMS practice opportunities using AR concepts such as embedding an accelerometer into a ball with direct feedback on the throw provided to children by colored LED’s (28), however, this study did not use location-based techniques. No studies have examined the impact of such games on children’s FMS development, despite the potential of these games in real world settings.

In summary, using AVG technology away from the constraints of the home environment may provide an opportunity for children to engage in physically active behaviors and practice and develop their FMS. Collaborations that use the skills of games designers and public health researchers could help to design mobile active games. Whilst research is needed to examine the efficacy of such techniques in real world settings, mobile active games may change our perspective of the potential of technology for enabling children’s physical activity and FMS.

## Conflict of Interest Statement

The authors declare that the research was conducted in the absence of any commercial or financial relationships that could be construed as a potential conflict of interest.

## Footnotes

^1^http://www.gosphero.com/tag/rolling-dead/

^2^https://itunes.apple.com/us/app/arsoccer-augmented-reality/id381035151?mt=8
